# Association of blood pressure variability and CT-based Leiden score in hypertension patients

**DOI:** 10.3389/fcvm.2023.1111120

**Published:** 2023-05-02

**Authors:** Jianqiao Chen, Xinqiang Ji, Runtao Zhao, Fan Wang

**Affiliations:** ^1^ Department of Geriatrics, Henan Provincial People's Hospital, People's Hospital of Zhengzhou University, People's Hospital of Henan University, Zhengzhou, China; ^2^ Department of Cardiology, The Second Medical Center & National Clinical Research Center for Geriatric Diseases, Beijing, China

**Keywords:** hypertension, blood pressure variability (BPV), coronary artery disease, coronary computed tomographic angiography (CCTA), Leiden score

## Abstract

**Background:**

Blood pressure variability (BPV) obtained from ambulatory blood pressure monitoring (ABPM) has been demonstrated to accurately predict the risk of cerebrovascular events and death in hypertension patients, however, the association between BPV and the severity of coronary atherosclerotic plaque remains unclear.

**Methods:**

Patients with hypertension combined with suspected coronary artery disease (CAD) were collected, who underwent both ABPM and coronary computed tomographic angiography (CCTA) from December 2017 to March 2022. Patients were divided into three groups according to the Leiden score: low-risk group (Leiden score <5), medium-risk group (Leiden score 5–20), and high-risk group (Leiden score >20). The clinical characteristics of patients were collected and analyzed. Univariate Pearson correlation and multivariate Logistics regression were used to determine the association between BPV and the severity of coronary atherosclerotic plaque.

**Results:**

A total of 783 patients were included, with the average age of (62.85 ± 10.17) years and 523 males. Patients in the high-risk group had higher mean systolic blood pressure (SBP), nighttime mean SBP and SBP variability (*P* < 0.05). Leiden score with low risk was associated with 24 h-SBP variability (*r* = 0.35, *P* = 0.006) and 24 h-diastolic blood pressure (DBP) loading (*r* = −0.18, *P* = 0.027). Leiden score with medium and high risk was associated with nighttime mean SBP (*r* = 0.23, *P* = 0.005), 24 h-SBP variability (*r* = 0.32, *P* = 0.003), and the decrease of nighttime SBP (*r* = 0.24, *P* = 0.019). Multivariate Logistic analysis showed that smoking [odds ratio (OR) = 1.014, 95% confidential interval (CI): 1.0–1.07, *P* = 0.03], diabetes (OR = 1.43, 95% CI: 1.10–2.26, *P* = 0.01) and 24 h-SBP variability (OR = 1.35, 95% CI: 1.01–2.46, *P* = 0.01) were independently associated with Leiden score with medium and high risk.

**Conclusion:**

Larger SBP variability in hypertensive patients indicates the higher Leiden score and consequently the more serious coronary atherosclerotic plaque. Monitoring SBP variability has certain significance for predicting the severity of coronary atherosclerotic plaque and preventing its progression.

## Introduction

Coronary artery disease (CAD) is a significant public health concern worldwide, with increasing mortality and morbidity rates in recent years (1). Hypertension is a known predictor of CAD incidence and development, and some hypertensive patients have a stronger association with CAD (2). Ambulatory blood pressure monitoring (ABPM) is an essential tool for assessing CAD risk and guiding personalized antihypertensive therapy (3, 4). Blood pressure variability (BPV), measured through ABPM or visit-to-visit assessments, has been shown to correlate with CAD incidence and development (5, 6). However, the impact of BPV on the severity of coronary atherosclerotic plaque remains unclear.

**Figure 1:**
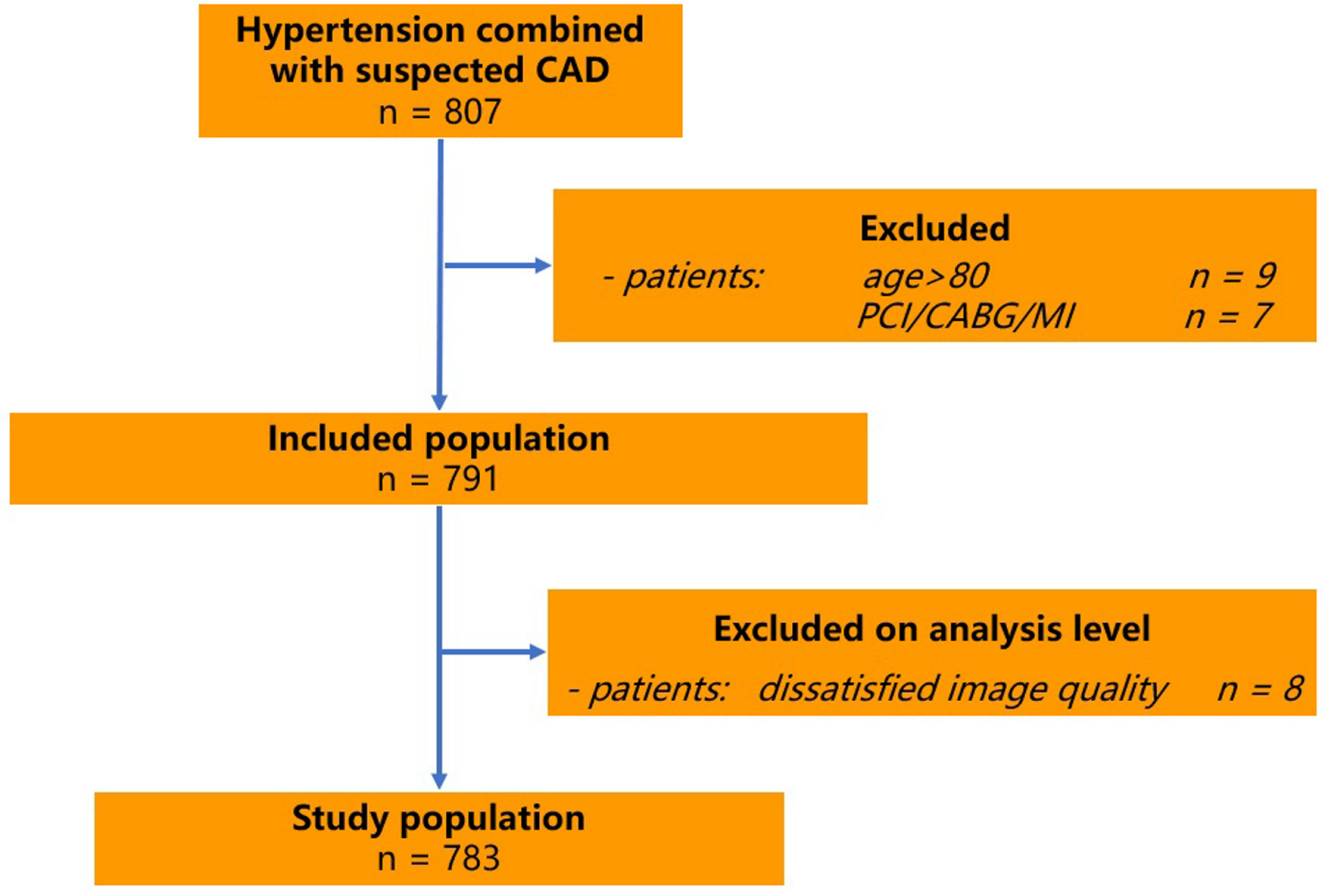
Protocol of study.

**Figure 2:**
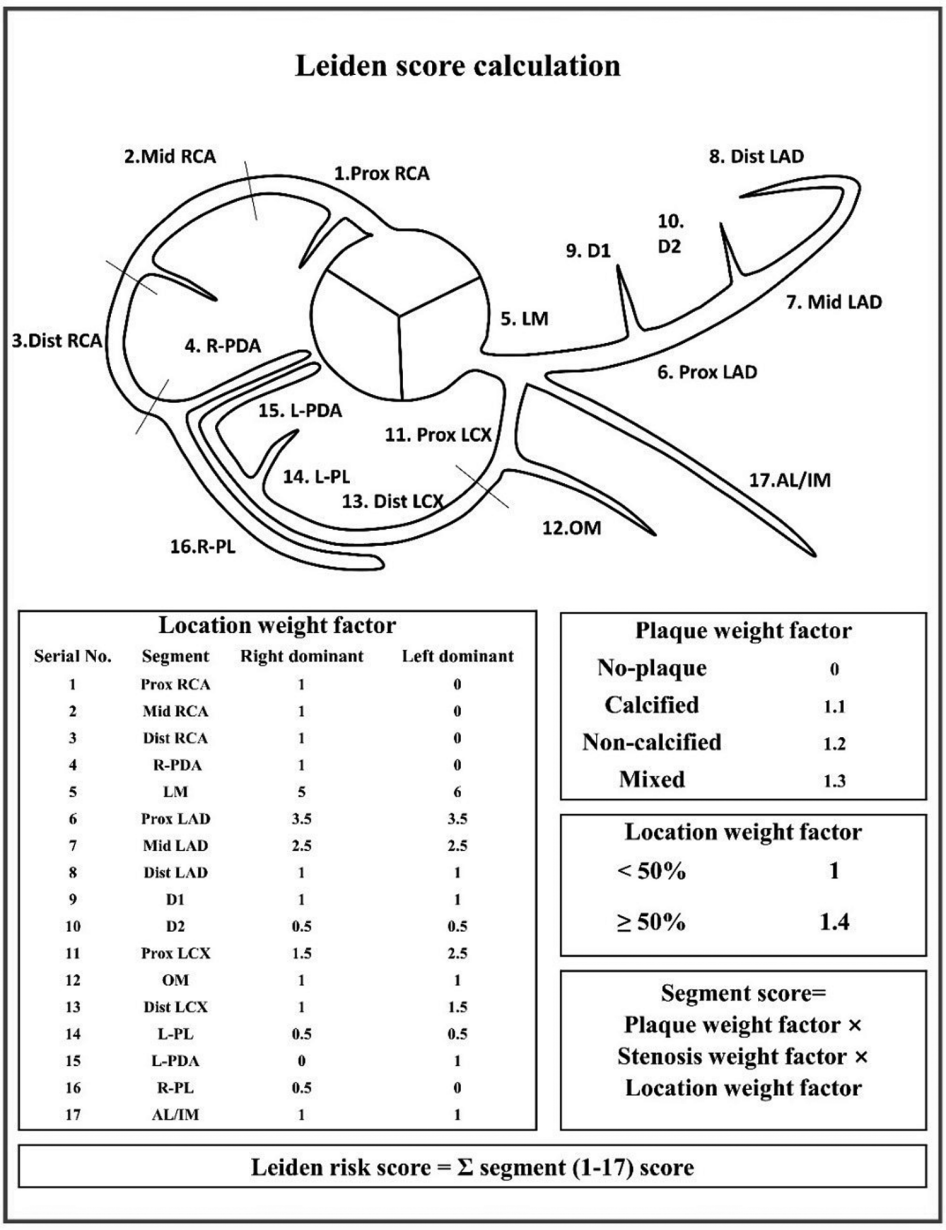
The specific scoring method of Leiden core.

Coronary computed tomographic angiography (CCTA) provides a non-invasive and comprehensive evaluation of the severity of coronary atherosclerotic plaque (7). The Leiden score, derived from CCTA, utilizes indicators related to the location, composition, and stenosis of plaque and has been shown to effectively predict long-term cardiovascular events (8). This study aims to investigate the relationship between blood pressure variability (BPV) and the severity of coronary atherosclerotic plaque as determined by the Leiden score.

## Materials and Methods

### Subjects

Patients with hypertension and suspected CAD who underwent ABPM and CCTA at Chinese PLA General Hospital between December 2017 and March 2022 were consecutively included in this study. The inclusion criteria were individuals (i) aged 18–80 years old with (ii) image quality that met qualitative and quantitative assessment standards, and (iii) who underwent both ABPM and CCTA within a month. Exclusion criteria were patients with cardiac arrhythmias, known hypersensitivity to iodine contrast media, or pregnancy.

Patients with an uninterpretable CTA examination, previous percutaneous intervention, coronary artery bypass surgery or myocardial infarction were excluded (Figure 1). Height and weight were measured and recorded, and subsequently BMI was calculated as weight in kilograms divided by the height in meters squared (kg/m^2^). The investigation was completed by physicians in the Department of Geriatric Cardiology of the PLA General Hospital who were trained by the research team. The diagnostic criteria for hypertension were referred to the World Health Organization Guideline for Hypertension Pharmacological Treatment in Adults (9). This study was performed according to the Declaration of Helsinki regarding investigations in humans and approved by the ethics committee of the Chinese PLA General Hospital. Written informed consent was obtained from each patient.

### Laboratory measurements

Blood sampling was performed between 7:30 AM and 8:30 AM after overnight fast after admission to hospital. Serum levels of total cholesterol, triglycerides, high density lipoprotein cholesterol (HDL cholesterol), plasma glucose, creatinine and uric acid were measured by a qualified technician using enzymatic assays (Roche, Basel, Switzerland) with a full automatic biochemical autoanalyser (COBAS c6000, Roche). Low-density lipoprotein cholesterol (LDL cholesterol) was calculated using the Friedewald formula. Renal function was estimated *via* eGFR. eGFR (ml/min/1.73 m^2^)= 175 × standard creatinine (mg/dl)^−1234^ × age (year)^−0.179^ × 0.79 (if female).

### Definition of variables

Cigarette smoking was defined as smoking one cigarette per day and for a duration of at least 1 year. Hypertension was indicated by the following: (i) systolic blood pressure (SBP) ≥140 mmHg; (ii) diastolic blood pressure (DBP) ≥90 mmHg; and/or (iii) the use of an antihypertensive drug (6). All participants without a history of diabetes mellitus were given a standard 75 g oral glucose tolerance test (OGTT). Fasting venous blood was collected from participants with a history of diabetes mellitus to measure blood glucose. Diabetes mellitus was indicated by (i) a fasting glucose level ≥7.1 mmol/L, (ii) a 2 h venous blood glucose level ≥11.1 mmol/L, or (iii) the use of a hypoglycemic drug or insulin (10).

### ABPM

All BP measurements using a noninvasive portable BP monitor (DM Corporation, model DMS-ABP2, USA) in the same arm in the sitting position after at least 5 min of rest. ABPM parameters included mean systolic BP (SBP), mean diastolic BP (DBP), mean daytime SBP, mean daytime DBP, mean nighttime SBP, mean nighttime DBP, decrease in nighttime BP (%), morning peak BP, BPV, and BPV coefficient. morning peak BP = mean SBP within 2 h after getting up - the lowest value of SBP during sleep at night (including the lowest value, the average value of three times before and after 1 h). BPV = standard deviation of 24 h BP. BPV coefficient = BPV divided by the mean 24 h BP (11).

### CCTA acquisition and image analysis

Routine CCTA using dual-source computed tomography (DSCT) scanner (Definition Flash, Siemens Healthcare, Germany) was performed in accordance with societal guidelines (12). Pre-treatment with beta-blockers was administrated if necessary, targeting a heart rate <60 beats/min. Sublingual nitrates were given to all patients before scanning. CCTA was done after injection of 50–90 ml iodine contrast *via* an ante cubital vein. Experienced local site investigators assessed luminal diameter stenosis in each segment of the coronary arteries. Plaque properties were defined as calcified plaque, non-calcified plaque, and mixed plaque according to relevant guidelines (13, 14). Coronary segment with diameters >1.5 mm was evaluated, and the corresponding stenosis grade (coronary artery disease-reporting and data system, CAD-RADS) was given at the patient level according to the diameter stenosis rate (15).

### Quantitative score of CCTA

The Leiden score assigns weight to different degree of lesion location, plaque composition and stenosis. Segment score is calculated as the multiplication of the weight factors for lesion location, plaque, and stenosis. The final score, that is, Leiden score, is calculated by addition of the individual segment scores, with higher scores indicating more severe coronary atherosclerotic plaque lesions (8). The specific scoring method is shown in the Figure 2.

### Statistical analysis

Values are expressed as mean ± SD if the variable was normally distributed, or median (interquartile range) if not. The Shapiro–Wilk test was used to assess whether data were normally distributed or not. A two-tailed *P* < 0.05 was considered statistically significant.

Patients were divided into three groups according to the Leiden score level: low-risk group (Leiden score <5), medium-risk group (Leiden score 5–20), and high-risk group (Leiden score >20). Groups were compared using the Student's *t*-test or Mann–Whitney *U* test for continuous values, and the *χ*^2^ test for categorical data, as appropriate.

Then patients were divided into two groups according to Leiden score level: low-risk group (Leiden score <5), medium and high-risk group (Leiden score ≥5). The univariate and multivariate correlation analysis of ABPM parameters and Leiden score was performed. One-way Spearman correlation and multivariate Logistic correlation were used to analyze the association between BPV and Leiden score. Data entry and management were undertaken with Microsoft Excel (Microsoft, Seattle, WA, USA) spreadsheet. All statistical analyzes were performed with SPSS 22.0 software (IBM Corporation, Armonk, NY, USA) was used to perform statistical analyses. *P* < 0.05 was considered statistically significant.

## Results

### Patient characteristics

The patient characteristics in all patients low-risk, medium-ris, and high-risk groups are shown in Table 1. The mean age of the study population was 62.85 ± 10.17 years, 523 (66.8%) were male, and the age, history of CAD, history of dyslipidemia, and the usage of statins were significantly different among the three groups (*P *< 0.05). As shown in Table 1, compared with patients in the low-risk group, those in the high-risk group were more prevalent in smoking (33.7% vs. 42.1%, *P *< 0.05) and had higher levels of cholesterol (4.24 vs. 4.56 mmol/L, *P *< 0.05).

**Table 1:** Patient characteristics across the different groups of Leiden score.

Clinical characteristics	All (*N* = 783)	Low risk (*N* = 254)	Medium risk (*N* = 340)	High risk (*N* = 189)
Age (years)	62.85 ± 10.17	47.76 ± 6.09	64.38 ± 4.45	72.76 ± 6.12^┼^
Sex (male)	523 (66.8%)	174 (68.5%)	207 (60.9%)*	142 (75.7%)*
BMI (kg/m^2^)	25.29 ± 3.37	25.47 ± 3.44	25.10 ± 3.09	25.31 ± 2.65
Smoking, *n* (%)	267 (34.1%)	72 (28.5%)	115 (33.7%)	80 (42.1%)*
CAD, *n* (%)	318 (65.7%)	95 (45.9%)	223 (79.5%)	189 (96%)^┼^
Dyslipidemia, *n* (%)	530 (67.8%)	135 (53.1%)	235 (69.4%)	147 (77.8%)^┼^
Diabetes mellitus, *n* (%)	111 (14.1%)	29 (11.4%)	50 (14.7%)	32 (16.9%)
Stroke/TIA, *n* (%)	38 (4.8%)	11 (4.4%)	19 (5.7%)	8 (4.3%)
CAD family history, *n* (%)	122 (15.6%)	37 (14.6%)	53 (15.6%)	32 (16.9%)
FBG (mmol/L)	5.68 (4.16–6.92)	5.41 (4.78–6.69)	5.82 (5.07–6.88)	5.48 (4.86–6.74)
TC (mmol/L)	4.15 ± 0.83	3.76 ± 0.65	4.24 ± 0.76	4.56 ± 0.82*
TG (mmol/L)	1.37 (0.94–1.91)	1.36 (0.96–1.92)	1.45 (0.86–1.85)	1.37 (1.03–2.84)
HDL-C (mmol/L)	1.24 (1.03–1.47)	1.33 (1.00–1.66)	1.23 (1.03–1.41)	1.19 (0.93–1.32)
LDL-C (mmol/L)	2.46 ± 0.71	2.59 ± 0.71	3.37 ± 0.70*	2.65 ± 0.86
UA (µmol/L)	349.55 ± 85.27	334.30 ± 68.22	352.21 ± 95.47	335.80 ± 75.46
eGFR (ml/min/1.73 m^2^)	90.37 (79.22–98.59)	96.13 (82.92–103.62)	89.68 (80.22–97.15)	85.10 (79.41–91.24)
Statins	193 (28.9%)	32 (12.6%)	111 (32.6%)	83 (43.9%)^┼^
Antihypertensives	703 (89.8%)	230 (90.2%)	308 (90.6%)	165 (87.0%)

^┼^ *P *< 0.05, the three groups were compared.

* *P *< 0.05, compared with the low-risk group. BMI, body mass index; CAD, coronary artery disease; TIA, transient ischemic attack; FBG, fasting blood glucose; TC, total cholesterol; TG, triglyceride; HDL-C, high density lipoprotein cholesterol, LDL-C, low density lipoprotein cholesterol; UA, uric acid; eGFR, estimated glomerular filtration rate.

### The comparison of Bp parameters and Bp variability in the different Leiden score level

Patients' results of ABPM are shown in Table 2. 24-hour mean SBP, nighttime mean SBP, nighttime mean DBP, and SBP variability differed significantly among the three groups (*P *< 0.05), and Leiden score increased with higher levels of the above ABPM parameters. Compared with the patients in low-risk group, those in medium-risk group showed the larger decrease of 24-hour mean SBP (34.57 vs. 39.66 mmHg, *P *< 0.05) and nighttime SBP (1.55 vs. 5.20 mmHg, *P *< 0.05).

**Table 2:** 24 h-ABPM parameters across the different groups of Leiden score.

24 h-ABPM parameters	All (*N* = 783)	Leiden score <5 (*N* = 254)	Leiden score 6–20 (*N* = 340)	Leiden score >20 (*N* = 189)
24 h-mean SBP (mmHg)	128.67 ± 12.95	127.69 ± 13.45	130.40 ± 12.54	134.80 ± 14.29^┼^
24 h-mean DBP (mmHg)	68.69 ± 8.54	69.93 ± 9.27	68.77 ± 7.84	70.35 ± 10.97
Daytime mean SBP (mmHg)	138.4 (113.0–149.0)	137.5 (119.0–148.0)	140.0 (114.0–146.0)	141.0 (120.0–168.0)
Daytime mean DBP (mmHg)	72.6 (67.0–84.0)	72.8 (61.3–81.5)	72.0 (66.50–82.0)	73.0 (69.50–86.0)
Nighttime mean SBP (mmHg)	131.5 (111.0–143.5)	129.5 (114.0–123.5)	135.0 (119.0–148.0)	152.50 (136.0–161.8)^┼^
Nighttime mean DBP (mmHg)	77.7 (52.3–99.3)	70.5 (62.75–83.50)	78.0 (71.0–90.60)	84.0 (70.0–101.2)^┼^
24 h-SBP variability (mmHg)	13.93 ± 3.23	12.74 ± 3.41	14.08 ± 3.09	16.42 ± 3.27^┼^
24 h-DBP variability (mmHg)	8.26 ± 3.55	8.56 ± 4.88	8.03 ± 2.02	8.31 ± 2.13
24 h-SBP loading value	37.48 ± 26.22	34.57 ± 27.70	39.66 ± 24.89*	36.83 ± 25.89
24 h-DBP loading value	0.3 (0.00–4.50)	0 (0.00–5.00)	0.7 (0.00–4.00)	0.2 (0.00–4.00)
Decrease of nighttime SBP (mmHg)	2.91 (−6.53–8.08)	1.55 (−5.05–7.43)	5.20 (−3.60–10.40)*	0.80 (−7.25–6.80)
Decrease of nighttime DBP (mmHg)	1.96 (−4.05–8.23)	4.05 (−3.60–10.80)	4.90 (−1.80–12.20)	3.40 (−5.45–9.85)

^┼^ *P *< 0.05, the three groups were compared.

* *P *< 0.05, compared with the low-risk group. ABPM, ambulatory blood pressure monitoring; DBP, diastolic blood pressure; SBP, systolic blood pressure.

### Associations between clinical data and Leiden score

The correlations between clinical data and Leiden score as assessed by univariate regression analysis are shown in Table 3. In the low-risk group, SBP variability (*r *= 0.35, *P *= 0.006) and DBP loading (*r *= −0.18, *P *= 0.027) were associated with Leiden score. In the medium- and high-risk group, mean nighttime SBP (*r *= 0.23, *P *= 0.005), SBP variability (*r *= 0.32, *P *= 0.003) and the decrease of nighttime SBP (*r *= 0.24, *P *= 0.019) were correlated with the Leiden score. Table 4 shows the results of a multivariate regression analysis to identify the factors associated with Leiden score. After adjustment for age, gender, BMI, FBG, UA, etc., multivariate Logistic analysis showed that smoking, history of diabetes, and SBP variability were associated with Leiden score in the medium- and high-risk group.

**Table 3:** Univariate correlation analysis between 24 h-ABPM parameters and Leiden score.

24 h-ABPM parameters	Leiden score <5 (*N* = 254)		Leiden score ≥5 (*N* = 529)	
	*r*	*P*-value	*r*	*P*-value
24 h-mean SBP (mmHg)	0.04	0.619	0.08	0.151
24 h-mean DBP (mmHg)	−0.07	0.426	0.09	0.119
Daytime mean SBP (mmHg)	0.06	0.464	0.09	0.089
Daytime mean DBP (mmHg)	−0.08	0.313	0.06	0.304
Nighttime mean SBP (mmHg)	0.05	0.336	0.23	0.005^┼^
Nighttime mean DBP (mmHg)	0.02	0.792	0.03	0.602
24 h-SBP variability (mmHg)	0.35	0.006^┼^	0.32	0.003^┼^
24 h-DBP variability (mmHg)	−0.12	0.134	0.06	0.315
24 h-SBP loading value	0.02	0.852	0.04	0.418
24 h-DBP loading value	−0.18	0.027^┼^	0.01	0.893
Decrease of nighttime SBP (mmHg)	−0.15	0.059	0.24	0.019^┼^
Decrease of nighttime DBP (mmHg)	−0.10	0.235	0.04	0.445

^┼^ *P*-value <0.05 is considered statistically significant. ABPM, ambulatory blood pressure monitoring; DBP, diastolic blood pressure; SBP, systolic blood pressure.

**Table 4:** Univariate and multivariate correlation analysis between clinical characteristics and Leiden score.

Clinical characteristic	Univariable		Multivariable	
	OR	*P*	OR	*P*
Age (years)	0.91 (0.66–1.26)	0.17	–	–
Sex	1.47 (0.67–3.23)	0.34	–	–
BMI	0.99 (0.95–1.03)	0.51	–	–
Smoking	1.04 (1.01–1.08)	0.02^┼^	1.014 (1.0–1.07)	0.03^┼^
Dyslipidemia	1.40 (0.63–3.10)	0.41	–	–
Diabetes	1.34 (1.04–2.09)	0.01^┼^	1.43 (1.10–2.26)	0.01^┼^
Stroke/TIA	1.77 (0.31–10.13)	0.52	–	–
CAD family history	0.83 (0.31–2.20)	0.71	–	–
FBG (mmol/L)	1.27 (0.84–1.93)	0.26	–	–
TC (mmol/L)	1.02 (0.73–1.44)	0.90	–	–
TG (mmol/L)	1.13 (0.83–1.54)	0.43	–	–
LDL-C (mmol/L)	0.88 (0.55–1.40)	0.59	–	–
HDL-C (mmol/L)	0.78 (0.24–2.51)	0.68	–	–
UA (umol/L)	0.99 (0.99–1.00)	0.59	–	–
eGFR (ml/min/1.73 m^2^)	0.99 (0.97–1.12)	0.58	–	–
24 h-mean SBP (mmHg)	1.02 (1.0–1.04)	0.04^┼^	0.99 (0.94–1.04)	0.71
24 h-mean DBP (mmHg)	0.99 (0.97–1.03)	0.92	–	–
Daytime mean SBP (mmHg)	1.02 (0.99–1.04)	0.07	–	–
Daytime mean DBP (mmHg)	0.99 (0.97–1.03)	0.85	–	–
Nighttime mean SBP (mmHg)	1.02 (1.0–1.04)	0.02^┼^	1.02 (0.97–1.07)	0.42
Nighttime mean DBP (mmHg)	0.99 (0.97–1.03)	0.97	–	–
24 h-SBP variability (mmHg)	1.17 (1. 05–1.31)	0.006^┼^	1.35 (1.01–2.46)	0.01^┼^
24 h-DBP variability (mmHg)	1.02 (0.91–1.15)	0.73	–	–
24 h-SBP loading value	1.01 (1.0–1.02)	0.13	–	–
24 h-DBP loading value	1.0 (0.99–1.01)	0.99	–	–
Decrease of nighttime SBP (mmHg)	0.97 (0.93–1.01)	0.15	–	–
Decrease of nighttime DBP (mmHg)	0.99 (0.97–1.03)	0.86	–	–
With antihypertension therapy	1.46 (0.68–3.15)	0.33	–	–
With anti-dyslipidemia therapy	0.88 (0.74–0,91)	0.02^┼^	0.93 (0.92–1.05)	0.68

Stepwise regression analysis is used to investigate the association between Leiden score and clinical parameters. Data are shown after adjustment for age, sex, BMI, FBG, UA, etc. β, regression coefficient.

^┼^ *P* < 0.05 with statistical significance. BMI, body mass index; CAD, coronary artery disease; TIA, transient ischemic attack; FBG, fasting blood glucose; TC, total cholesterol; TG, triglyceride; HDL-C, high density lipoprotein cholesterol; LDL-C: low density lipoprotein cholesterol; UA, uric acid; eGFR, estimated glomerular filtration rate.

## Discussion

In this study, we explored the association of BPV and coronary atherosclerotic plaque severity in patients with hypertension. We found that 24-hour mean SBP, nighttime mean SBP, nighttime mean DBP, and SBP variability were positively correlated with Leiden score. Univariate correlation analysis between ABPM parameters and Leiden score showed that the more significant SBP fluctuation and the higher nighttime SBP were associated with more severe coronary atherosclerotic plaque lesions. Further multivariate correlation analysis showed that SBP variability, smoking and history of diabetes were associated with the medium- and high-risk Leiden score, indicating that the more significant SBP fluctuations were associated with more severe coronary atherosclerotic plaque.

As ABPM continuously gains prevalence and progress in clinical practice, several studies have found that ABPM not only describe trends in BP fluctuations, but also access association with CAD. Rothwell PM et al. (2) found that BPV are strong predictors of stroke, independent of mean SBP, and increased BPV in patients with treated hypertension is associated with a high risk of vascular events. Cremer et al. (4) followed up 969 hypertension patients who underwent ABPM and found that BPV was significantly associated with the incidence of adverse cardiovascular events, including acute coronary syndrome, ischemic or hemorrhagic stroke, aortic coarctation, and abdominal aortic aneurysm. Study of Wei et al. (5) analyzed the patients with heart failure with preserved ejection fraction and showed that BPV (Visit-to-Visit) was associated with poor prognosis consisting of cardiovascular death, heart failure hospitalization, and stroke. A meta-analysis conducted by Stevens et al. (16) showed that long-term increase in SBP variability was significantly associated with all-cause mortality, cardiovascular disease (CVD) mortality, and the incidence of CVD, CAD, and stroke. Based on above findings, the role of BPV as a possible indicator for the evaluation of coronary plaque progression has attracted doctors' attention and entered the clinical practice. However, the relationship between BPV and the severity of coronary atherosclerotic plaque lesions was not discussed in the aforementioned studies. Previous studies showed that BPV correlated with the volume of coronary atherosclerotic plaque in patients with CAD. Aoyama et al. (17) retrospectively collected 36 patients with both hypertension and stable CAD. These patients underwent ABPM and optical coherence tomography-guided coronary angiography, and then the core size and the fibrous cap thickness of the plaque were measured and analyzed. The results showed that BPV was significantly correlated with the volume of coronary atherosclerotic plaques, indicating that patients with larger BPV were more likely to develop atherosclerotic plaques. Nevertheless, it should be noted that this study had small sample size, besides, it used invasive assessment method, which was expensive and complex to measure, and was difficult to be duplicated in clinical practice. Therefore, unlike previous studies, the present study utilized the non-invasive and accurate CCTA-based Leiden score to evaluate coronary atherosclerotic plaque loading, which has the advantages of short examination time (10–15 min), high accuracy, and generally acceptable examination cost (between 800 and 1,300 RMB), and is now widely used in the clinical practice of CAD (1). A study including 2,809 patients showed that compared with risk groups based on stenosis severity alone, Leiden score could provide more comprehensive evaluation of coronary atherosclerotic plaque composition, location, and stenosis, suggesting that Leiden score has better prognostic ability and exhibit advantages in risk stratification of coronary plaque loading (8).

The present study not only adopted the Leiden score with the superior clinical utility to evaluate the severity of coronary atherosclerotic plaque lesion, but also further clarified the clinical significance of BPV, that is, the significant correlation between BPV and the severity of coronary atherosclerotic plaque. The traditional view is that dyslipidemia is one of the important influencing factors of coronary plaque progression, but in this study, TC/LDL-C is not associated with the Leiden score, there may be two reasons: The Leiden score cannot systematically assess high risk plaque features (8); Heterogeneity of population.

Our study has some limitations. The enrolled subjects were mainly elderly patients with more clinical combinations of CAD, diabetes and other underlying diseases, and the percentage of males (66.8%) was higher than that of females. Besides, it is a single-center and retrospective study, and the findings need to be validated in larger clinical studies.

In conclusion, our study found that the severity of coronary atherosclerotic plaque lesions tended to increase with the increment of SBP variability. SBP variability as an independent risk factor for coronary atherosclerosis requires further investigation, aiming to provide theoretical basis for the targeted BP treatment of halting or delaying the progression of coronary atherosclerotic plaques and preventing cardiovascular events. Based on the traditional cardiovascular risk factors, further risk stratification of CAD based on BPV is undoubtedly of great clinical significance and health economics value to improve the prognosis of CAD patients.

## Data Availability

The raw data supporting the conclusions of this article will be made available by the authors, without undue reservation.
